# A rare case of mammary hamartoma presenting as malignant on radiological assessment and benign on pathological examination: a case report

**DOI:** 10.3389/fonc.2025.1593952

**Published:** 2025-09-10

**Authors:** Qingfeng Yang, Yiping Gong, Jin Hu

**Affiliations:** Department of Breast and Thyroid Surgery, Renmin Hospital, Wuhan University, Wuhan, China

**Keywords:** breast, hamartoma, case report, breast hamartoma, mammary hamartoma

## Abstract

Breast hamartomas are rare, benign, and encapsulated lesions composed of a combination of fatty, glandular, muscular, and fibrous tissue. Mammography provides an overview of the breast’s structure and can identify the characteristic “breast within a breast” appearance typical of hamartomas. Ultrasound is useful for determining the echogenicity and vascularization of hamartomas, thereby helping to differentiate them from potential malignancies. Magnetic Resonance Imaging (MRI) is another indispensable tool in the diagnostic arsenal for breast hamartomas. One of the major challenges in differential diagnosis is distinguishing hamartomas from fibroadenomas, which typically occur in young women. Here, we present a case of a 21-year-old female with a highly suspicious lesion based on radiological features, which was ultimately diagnosed histologically as a breast hamartoma.

## Introduction

Breast hamartomas are uncommon, benign, and encapsulated lesions with unclear etiology and pathogenesis (1). They are characterized by an exceptionally low clinical incidence, accounting for approximately 4.8% of all benign breast masses (2). These lesions are composed of a mixture of glandular, fatty, fibrous, and muscular tissues (3). Although typically benign, their clinical presentation and diagnostic features pose unique challenges to clinicians, making their study significant for medical practice. Breast hamartomas usually occur in middle-aged, perimenopausal women but can develop at any age (3). Although these tumors are uncommon, they can grow to substantial sizes and may co-occur with malignant tumors. Surgical resection is the first-line treatment. In this report, we describe a rare case of a breast hamartoma that exhibited discordant pathological and radiological findings.

## Case presentation

A 21-year-old woman presented to our hospital with a palpable lump in her right breast. She reported that the mass had been present for approximately two months, during which it had gradually increased in size, accompanied by a mild discomfort but without significant pain or other symptoms. Her family medical history was unremarkable, and she denied any history of tobacco use. Physical examination revealed a painless, hard, ill-defined, poorly mobile mass in the upper lateral part of the right breast. A dimpling sign was observed in the breast.

**Figure 1:**
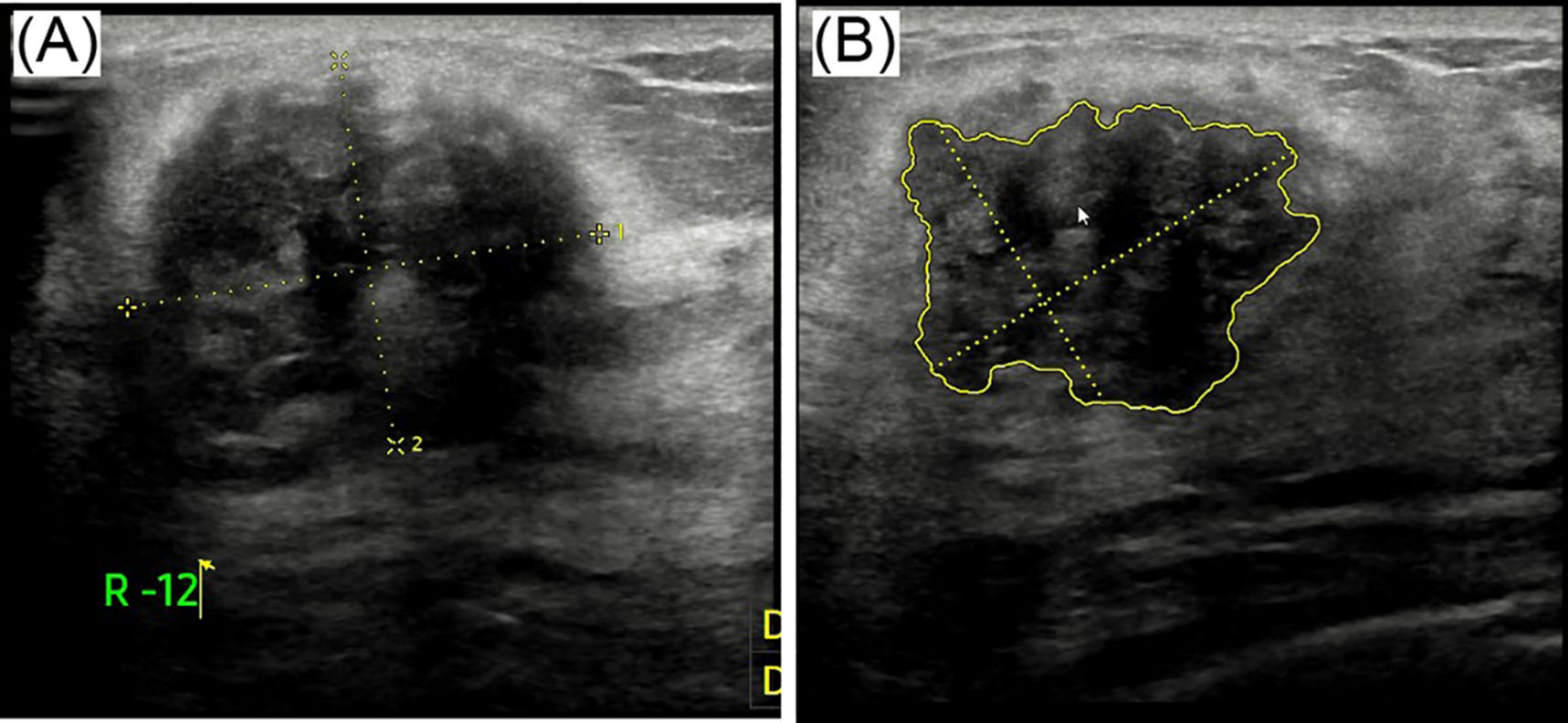
Ultrasound images labeled **(A)** and **(B)**. Image **(A)** shows a mass with dotted yellow lines indicating measurements. Image **(B)** highlights an irregularly shaped area outlined in yellow, also marked with measurement lines.

**Figure 2:**
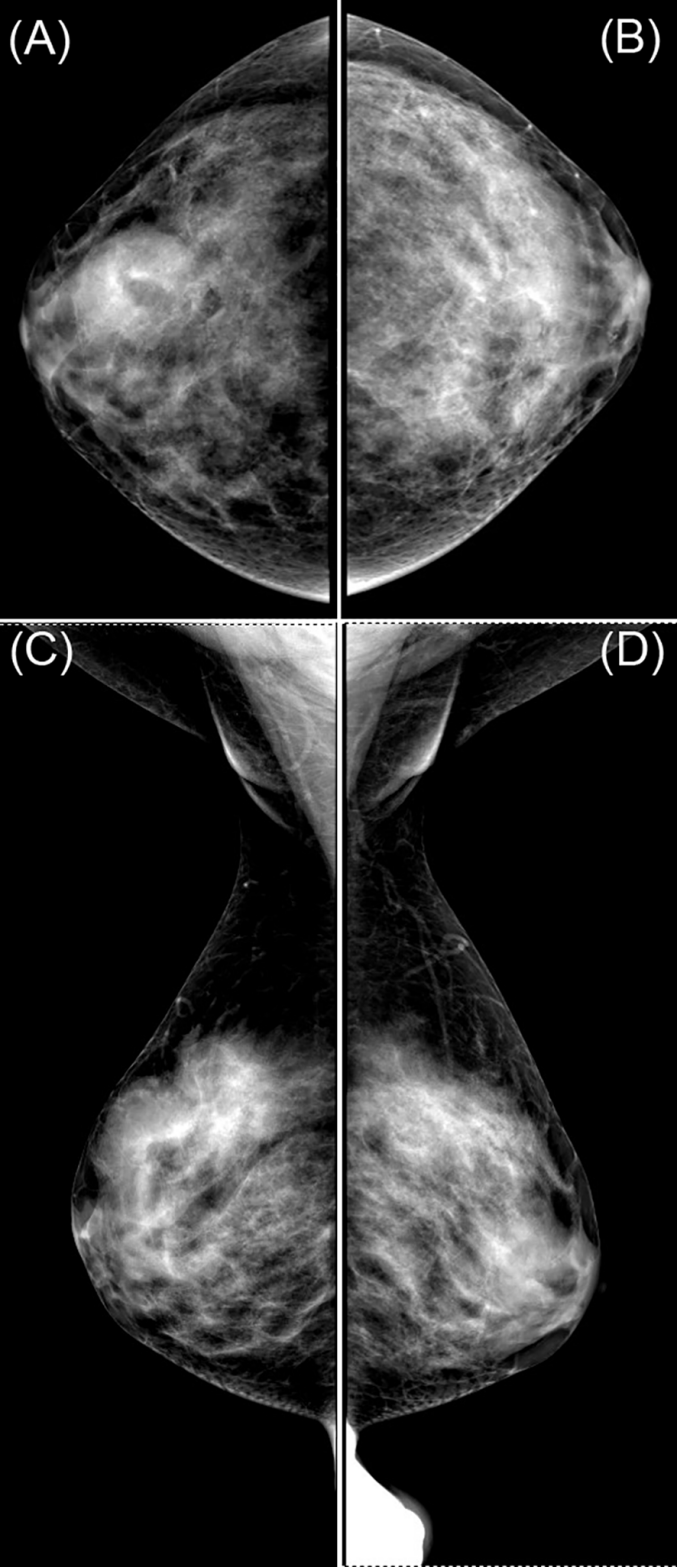
Mammogram images labeled **(A–D)** show different views of a breast, highlighting dense areas. Panels **(A, B)** are craniocaudal views, while **(C)** and **(D)** are mediolateral oblique views. The images display varying tissue densities, useful for medical analysis.

**Figure 3:**
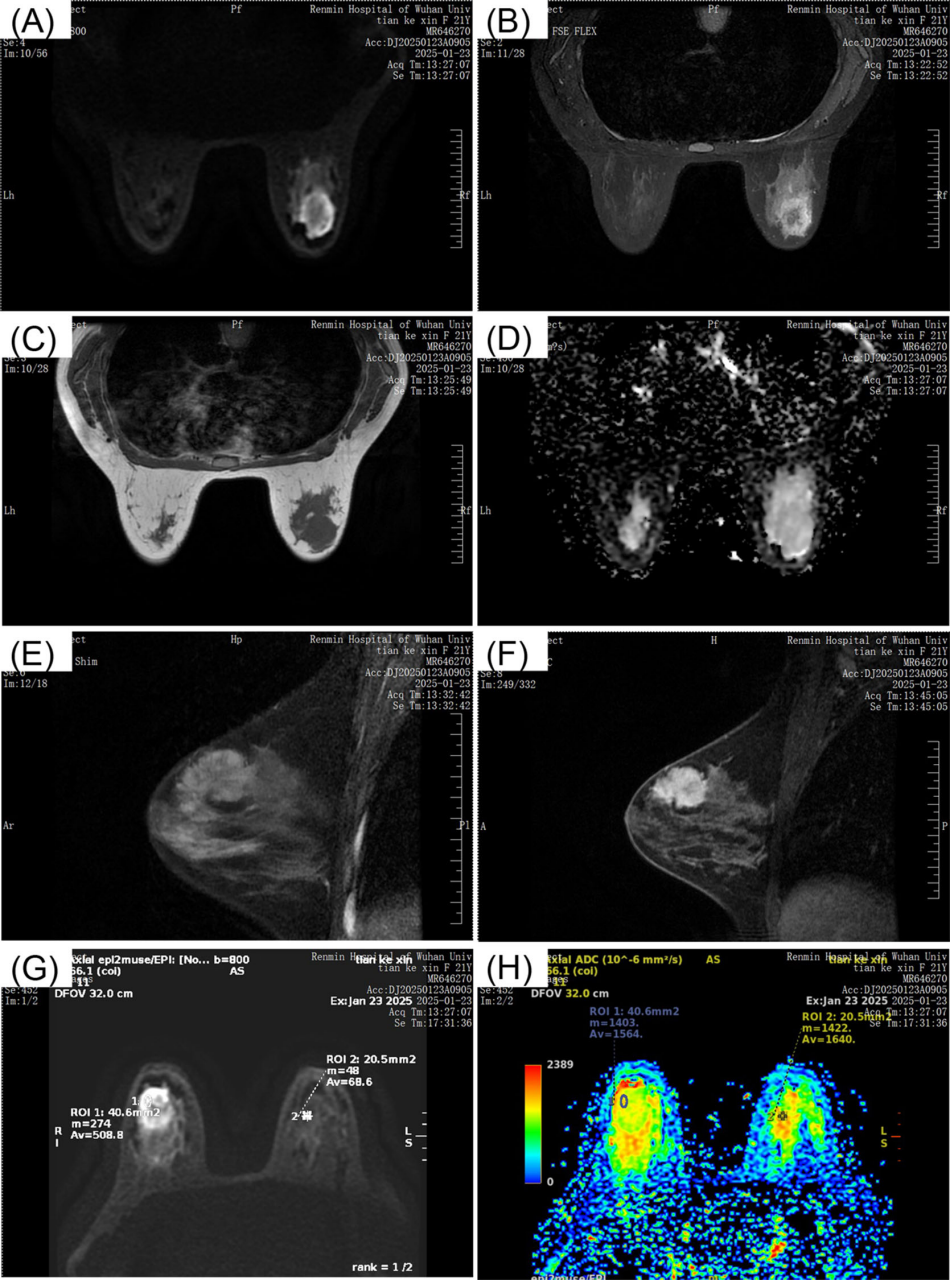
MRI breast scans depicting different views. Image **(A)** shows a coronal section with a visible mass. Image **(B)** presents another coronal view. Image **(C)** is a transverse section. Image **(D)** displays a diffusion-weighted image. Image **(E)** and **(F)** are sagittal views with noticeable lesions. Image **(G)** and **(H)** contain color maps indicating apparent diffusion coefficients, with regions of interest marked. All images are labeled with patient information and scan details from Renmin Hospital of Wuhan University, dated January 23, 2025.

**Figure 4:**
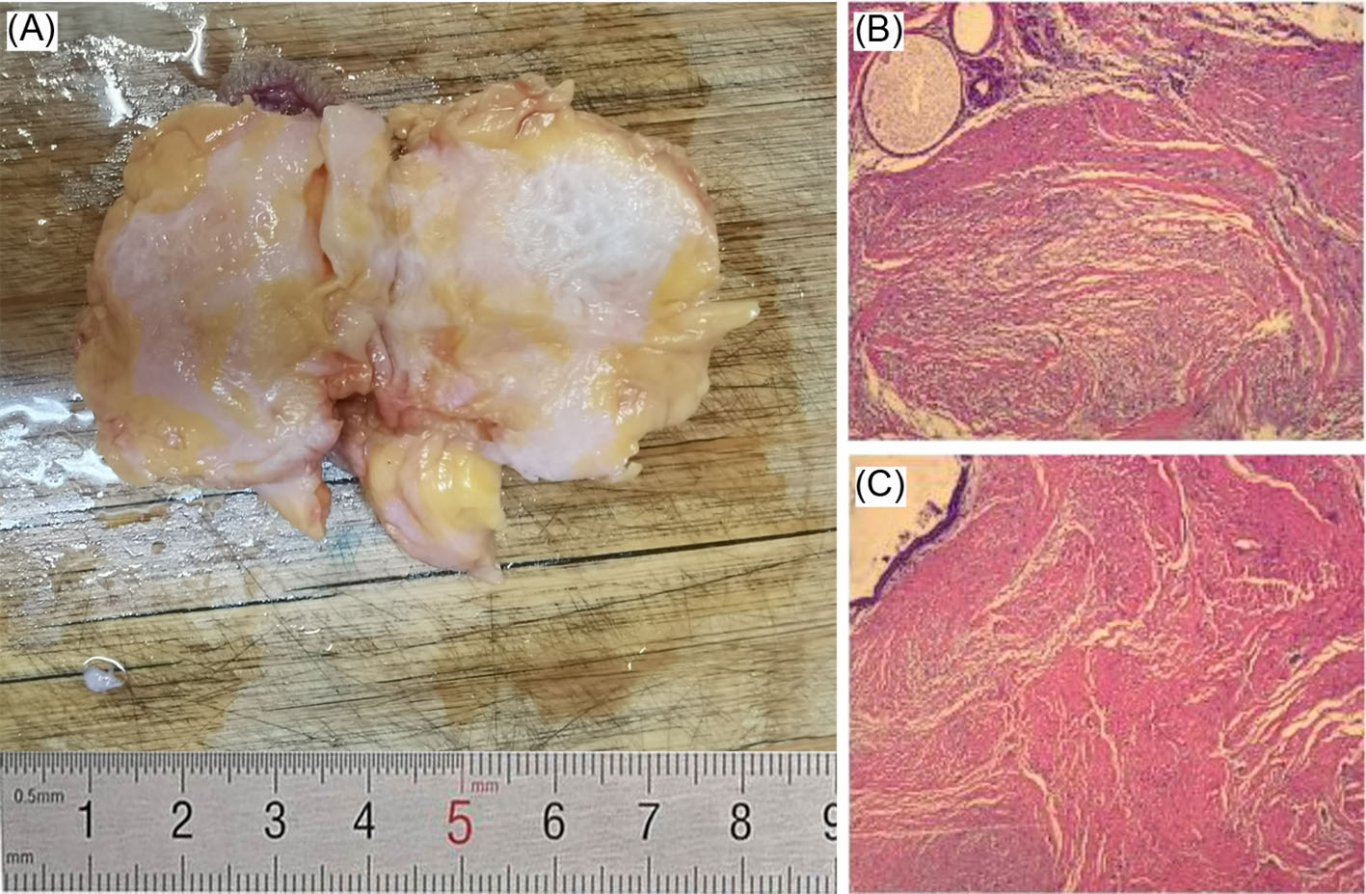
**(A)** Excised tissue sample on a cutting board with a ruler indicating size in centimeters. **(B)** and **(C)** Microscopic views of tissue sections displaying fibrous structures in shades of pink, red, and yellow.

Breast ultrasound revealed a hypoechoic mass in the right breast with indistinct borders, classified as BI-RADS IVc (**Figures 1A, B**). Bilateral mammography showed an oval, well-circumscribed, predominantly fatty mass measuring approximately 3.3×3.7 cm in the upper outer quadrant of the right breast (**Figure 2**), which was assigned a BI-RADS IVa score. Given the atypical appearance of the mass, further evaluation with magnetic resonance imaging (MRI) was performed. MRI demonstrated a mass-like lesion with mixed T1 signal and prolonged T2 signal in the upper quadrant of the right breast, measuring about 2.8 cm×3.1 cm×2.3 cm. The lesion exhibited heterogeneous enhancement during the contrast-enhanced scan (**Figure 3**), and a BI-RADS IVc score was reaffirmed. After discussion in our multidisciplinary team, the patient underwent surgical excision of the right breast mass. Fine-needle aspiration cytology (FNAC) was initially considered, but due to the patient’s young age and the clinical presentation of a mass with a dimpling sign, the multidisciplinary team opted for surgical excision to obtain a more definitive diagnosis and to address the patient’s concerns about potential malignancy. The patient underwent surgical excision of the right breast mass under general anesthesia, with careful dissection and removal of the mass while preserving the surrounding breast tissue. During the surgery, a rapid frozen section pathology was performed on the right breast mass. The frozen section pathology report indicated a benign lesion of the right breast, with a consideration of breast hamartoma. The interior of the resected tumor appeared yellow and white. Postoperative pathology revealed a mammary hamartoma in the right breast, measuring 3.5×3.3×3 cm. The tumor was well-defined and composed of randomly arranged glandular and stromal components, as well as adipose tissue and smooth muscle fibers (**Figure 4**). Immunohistochemistry results showed Desmin (focal +), ER (-), Ki-67 (+, approximately 5%), and SMA (+). The patient was followed up after three months, and an ultrasound report showed no recurrence.

## Discussion

The average age of patients with breast hamartomas ranges from 19 to 56 years, with a mean age of 41.8 years (4). Alran et al. reported a median age of 40 years (3). In our case, the patient was a 23-year-old young woman. Similarly, Aminpour N et al. reported a case of a 23-year-old female with myoid hamartoma of the breast (5). Therefore, when a young woman presents with a large, hard, slow-growing breast mass, and core-needle biopsy based on breast ultrasound suggests fibroadenoma, differentiation from breast hamartoma is necessary.

Breast hamartoma is rarely occurs in men. Gupta SS et al. reported a case in a 13-year-old boy (6).

Although breast hamartomas are generally slow-growing, the gradual growth observed in this case may be attributed to the patient’s young age and hormonal factors.

Hamartomas are rarely associated with malignancies. However, a few studies have reported invasive breast cancer coexisting with breast hamartoma. Sevim Y et al. identified invasive ductal carcinoma in one case and lobular carcinoma *in situ* in another (4). To our knowledge, there have been two reported cases of breast parenchymal hamartoma with synchronous contralateral breast cancer (7, 8). This association may be related to PTEN hamartoma tumor syndrome (PHTS), characterized by mutations in the PTEN tumor suppressor gene (8).

Mammary hamartoma is a relatively rare benign breast lesion composed of an abnormal mixture of adipose, glandular, and fibrous tissue, often forming a well-circumscribed mass. As slow-growing, benign entities, these lesions are distinct for their heterogeneous composition and generally favorable prognosis (1). Despite their benign nature, accurate recognition and diagnosis of mammary hamartomas are vital due to their potential to be confused with other, potentially malignant breast masses (9, 10). Such lesions are typically detected incidentally during routine breast imaging performed for other clinical indications.

Mammography is often the first-line imaging modality for evaluating breast lesions, including mammary hamartomas. Its ability to provide a detailed overview of the breast’s structure helps identify the classic “breast within a breast” appearance characteristic of hamartomas (11). However, mammography’s sensitivity is limited in dense breast tissue, where lesions can be obscured, making diagnosis challenging and often necessitating additional imaging.

Ultrasound is a non-invasive diagnostic tool that significantly supplements mammography by providing detailed information on the internal structure of breast masses. It offers real-time imaging and is particularly useful for differentiating solid from cystic lesions. Ultrasound also aids in assessing the echogenicity and vascularization of hamartomas, which can help distinguish them from malignancies (12, 13). Elastography, often combined with ultrasound, assesses tissue stiffness, a key feature differentiating benign from malignant lesions.

Magnetic Resonance Imaging (MRI) is another essential diagnostic tool for breast lesions. MRI offers high contrast resolution, making it ideal for imaging complex breast structures and revealing atypical vascular patterns (14). Its sensitivity to changes in tissue composition makes it a valuable adjunct when mammography and ultrasound results are inconclusive. Techniques like contrast-enhanced MRI can highlight regions with increased vascularity or unusual enhancement patterns suggestive of malignancy, thereby enhancing diagnostic accuracy (11, 15).

Clinically, hamartomas typically present as movable, well-circumscribed masses with a rubbery texture, similar to fibroadenomas (3).

In our clinical experience, the diagnostic prevalence of breast hamartoma appears lower than that reported in the literature. Diagnosis is typically established by core-needle biopsy combined with appropriate correlation of clinical and radiologic features. Breast hamartomas may be underdiagnosed because pathologists might categorize these lesions as fibroadenomas rather than hamartomas (4).

Differential diagnosis is crucial to ensure hamartomas are not misclassified, preventing potentially incorrect treatment. This requires integrated assessment of clinical, radiological, and histopathological data (16).

Differential diagnosis can be particularly challenging when distinguishing hamartomas from fibroadenomas, which share a similar imaging appearance (17). Both can appear as well-circumscribed, hypoechoic masses on ultrasound; however, hamartomas usually exhibit greater internal heterogeneity due to their composition of both fat and fibrous tissue (18). MRI is superior for delineating internal composition, enhancement patterns, and tissue characteristics compared to other modalities (12).

Management strategies for mammary hamartomas range from active surveillance to surgical excision, tailored to individual patient factors. Understanding the clinical relevance of treatment approaches is crucial, especially since many cases remain asymptomatic. While surgery is indicated in some cases, non-invasive management remains a viable alternative for most patients, underscoring the need for personalized treatment plans.

In summary, we describe an unusual case of breast hamartoma that presented with radiological features concerning for a highly malignant lesion. Surgical excision was the treatment of choice. Given the rarity of such presentations and the limited number of previously reported cases, this case provides valuable insights and warrants further investigation.

## Data Availability

The original contributions presented in the study are included in the article/supplementary material. Further inquiries can be directed to the corresponding authors.
